# ECDC’s latest publications

**DOI:** 10.2807/1560-7917.ES.2019.24.13.1903282

**Published:** 2019-03-28

**Authors:** Eurosurveillance editorial team

**Affiliations:** 1 European Centre for Disease Prevention and Control (ECDC), Stockholm, Sweden

**Keywords:** infectious diseases, public health, Europe

 

**Rapid risk and outbreak assessments**

 

Multi-country outbreak of Salmonella Poona infections linked to consumption of infant formula

Date of publication: 12 March 2019

https://ecdc.europa.eu/en/publications-data/rapid-outbreak-assessment-multi-country-outbreak-salmonella-poona-infections

 

Outbreak of VIM-producing carbapenem-resistant Pseudomonas aeruginosa linked to medical tourism to Mexico

Date of publication: 11 March 2019

https://ecdc.europa.eu/en/publications-data/rapid-risk-assessment-outbreak-vim-producing-carbapenem-resistant-pseudomonas

 

Rift Valley fever outbreak in Mayotte, France

Date of publication: 8 March 2019

https://ecdc.europa.eu/en/publications-data/rapid-risk-assessment-rift-valley-fever-outbreak-mayotte-france

 

Ebola virus disease outbreak in North Kivu and Ituri Provinces, Democratic Republic of the Congo – third update

Date of publication: 14 February 2019

https://www.ecdc.europa.eu/en/publications-data/risk-assessment-ebola-virus-outbreak-north-kivu-and-ituri-third-update

 

**Technical reports**

 

Operational tool on rapid risk assessment methodology - ECDC 2019

Date of publication: 14 March 2019

https://ecdc.europa.eu/en/publications-data/operational-tool-rapid-risk-assessment-methodology-ecdc-2019

 

Eighth external quality assessment scheme for typing of Shiga toxin-producing *Escherichia coli*

Date of publication: 28 February 2019

https://www.ecdc.europa.eu/en/publications-data/eighth-external-quality-assessment-scheme-typing-shiga-toxin-producing

 

Laboratory manual for carbapenem and colistin resistance detection and characterisation for the survey of carbapenem- and/or colistin-resistant Enterobacteriaceae

Date of publication: 18 February 2019

https://www.ecdc.europa.eu/en/publications-data/laboratory-manual-carbapenem-and-colistin-resistance-detection-and

 

European external quality assessment programme for influenza virus 2018

Date of publication: 11 February 2019

https://www.ecdc.europa.eu/en/publications-data/european-external-quality-assessment-programme-influenza-virus-2018

 

HIV Estimates Accuracy Tool manual

Date of publication: 31 January 2019

https://ecdc.europa.eu/en/publications-data/hiv-estimates-accuracy-tool-manual

 

Expert consensus protocol on colistin resistance detection and characterisation for the survey of carbapenem- and/or colistin-resistant Enterobacteriaceae

Date of publication: 14 January 2019

https://ecdc.europa.eu/en/publications-data/expert-consensus-protocol-colistin-resistance-detection-and-characterisation

 

Expert consensus protocol on carbapenem resistance detection and characterisation for the survey of carbapenem- and/or colistin-resistant Enterobacteriaceae

Date of publication: 14 January 2019

https://ecdc.europa.eu/en/publications-data/expert-consensus-protocol-carbapenem-resistance-detection-and-characterisation

 

External quality assessment for the detection of Bordetella pertussis by PCR, 2018

Date of publication: 11 January 2019

https://ecdc.europa.eu/en/publications-data/external-quality-assessment-detection-bordetella-pertussis-pcr-2018

 

Point prevalence survey of healthcare-associated infections and antimicrobial use in European acute care hospitals - ECDC PPS validation protocol version 3.1.2.

Date of publication: 10 January 2019

https://ecdc.europa.eu/en/publications-data/point-prevalence-survey-healthcare-associated-infections-and-antimicrobial-use-4

 

**Surveillance reports**

 

Tuberculosis surveillance and monitoring in Europe, 2019

Date of publication: 19 March 2019

https://ecdc.europa.eu/en/publications-data/tuberculosis-surveillance-and-monitoring-europe-2019

 

Monthly measles and rubella monitoring report, March 2019

Date of publication: 8 March 2019

https://ecdc.europa.eu/en/publications-data/monthly-measles-and-rubella-monitoring-report-march-2019

 

Gonococcal antimicrobial susceptibility surveillance in Europe, 2017

Date of publication: 28 February 2019

https://www.ecdc.europa.eu/en/publications-data/gonococcal-antimicrobial-susceptibility-surveillance-europe-2017

 

The European Union summary report on antimicrobial resistance in zoonotic and indicator bacteria from humans, animals and food in 2017

Date of publication: 26 February 2019

https://ecdc.europa.eu/en/publications-data/european-union-summary-report-antimicrobial-resistance-zoonotic-and-indicator-5

 

Monthly measles and rubella monitoring report, February 2019

Date of publication: 8 February 2019

https://www.ecdc.europa.eu/en/publications-data/monthly-measles-and-rubella-monitoring-report-february-2019

 

Influenza virus characterisation, Summary Europe, December 2018

Date of publication: 21 January 2019

https://ecdc.europa.eu/en/publications-data/influenza-virus-characterisation-summary-europe-december-2018

 

Monthly measles and rubella monitoring report, January 2019

Date of publication: 11 January 2019

https://ecdc.europa.eu/en/publications-data/monthly-measles-and-rubella-monitoring-report-january-2019

 

**Annual Epidemiological Report series on communicable diseases in Europe**

****

**Published in March:**

****

Chapter on Salmonellosis for 2016

https://ecdc.europa.eu/en/publications-data/salmonellosis-annual-epidemiological-report-2016

 

Chapter on West Nile virus infection for 2016

https://ecdc.europa.eu/en/publications-data/west-nile-virus-infection-annual-epidemiological-report-2016

 

Chapter on Creutzfeldt−Jakob disease for 2016

https://ecdc.europa.eu/en/publications-data/creutzfeldt-jakob-disease-annual-epidemiological-report-2016

****

**Published in February:**

 

Chapter on HIV infection and AIDS for 2017

https://www.ecdc.europa.eu/en/publications-data/hiv-infection-and-aids-annual-epidemiological-report-2017

 

Chapter on Rabies for 2017

https://www.ecdc.europa.eu/en/publications-data/rabies-annual-epidemiological-report-2017

 

Chapter on Rift Valley fever for 2017

https://www.ecdc.europa.eu/en/publications-data/rift-valley-fever-annual-epidemiological-report-2017

 

Chapter on trichinellosis for 2017

https://www.ecdc.europa.eu/en/publications-data/trichinellosis-annual-epidemiological-report-2017

 

Chapter on trichinellosis for 2016

https://www.ecdc.europa.eu/en/publications-data/trichinellosis-annual-epidemiological-report-2016

 

Chapter on plague for 2017

https://www.ecdc.europa.eu/en/publications-data/plague-annual-epidemiological-report-2017

 

Chapter on Crimean-Congo haemorrhagic fever for 2017

https://www.ecdc.europa.eu/en/publications-data/crimean-congo-haemorrhagic-fever-cchf-annual-epidemiological-report-2017

 

Chapter on malaria for 2016 

https://www.ecdc.europa.eu/en/publications-data/malaria-annual-epidemiological-report-2016

 

**Published in January:**

****

Chapter on zoonotic influenza for 2017 

https://ecdc.europa.eu/en/publications-data/zoonotic-influenza-annual-epidemiological-report-2017

 

Chapter on hepatitis A for 2016 

https://ecdc.europa.eu/en/publications-data/hepatitis-annual-epidemiological-report-2016

 

Chapter on Legionnaires’ disease for 2017 

https://ecdc.europa.eu/en/publications-data/legionnaires-disease-annual-epidemiological-report-2017

 

Chapter on cholera for 2017 

https://ecdc.europa.eu/en/publications-data/cholera-annual-epidemiological-report-2017

 

Chapter on tularaemia for 2016 

https://ecdc.europa.eu/en/publications-data/tularaemia-annual-epidemiological-report-2016

 

Chapter on congenital syphilis for 2017 

https://ecdc.europa.eu/en/publications-data/congenital-syphilis-annual-epidemiological-report-2017

 

Chapter on Q fever for 2016 

https://ecdc.europa.eu/en/publications-data/q-fever-annual-epidemiological-report-2016

 

Chapter on chlamydia infection for 2017 

https://ecdc.europa.eu/en/publications-data/chlamydia-infection-annual-epidemiological-report-2017

 

**Mission reports**

 

ECDC country visit to Norway to discuss antimicrobial resistance issues

Date of publication: 16 January 2019

https://ecdc.europa.eu/en/publications-data/ecdc-country-visit-norway-discuss-antimicrobial-resistance-issues

 

**Corporate publications**

****

Single programming document 2019–2021

Date of publication: 11 January 2019

https://ecdc.europa.eu/en/publications-data/single-programming-document-2019-2021

 

**ECDC communicable disease threats report**

Date of publication: every Friday

https://ecdc.europa.eu/en/threats-and-outbreaks/reports-and-data/weekly-threats

**

**Scientific peer-reviewed publications**

Publications by ECDC staff in scientific journals

Updated monthly

https://ecdc.europa.eu/en/publications-data?f%5b0%5d=output_types%3A1247

